# Indications for Heart Transplantation in Congenital Heart Disease

**DOI:** 10.2174/157340311797484240

**Published:** 2011-05

**Authors:** E Siân Pincott, M Burch

**Affiliations:** Department of Cardiology, Great Ormond Street Hospital, Great Ormond Street, London WCIN 3JH

**Keywords:** Congenital heart disease (CHD), heart transplantation, paediatric.

## Abstract

In this review we have looked at indications for cardiac transplantation in congenital heart disease. An outline of the general principles of the use of transplant as a management strategy both as a first line treatment and following other surgical interventions is discussed. We explore the importance of the timing of patient referral and the evaluations undertaken, and how the results of these may vary between patients with congenital heart disease and patients with other causes of end-stage heart failure. The potential complications associated with patients with congenital heart disease need to be both anticipated and managed appropriately by an experienced team. Timing of transplantation in congenital heart disease is difficult to standardize as the group of patients is heterogeneous. We discuss the role and limitations of investigations such as BNP, 6 minute walk, metabolic exercise testing and self estimated physical functioning. We also discuss the suitability for listing. It is clear that congenital heart patients should not be considered to be at uniform high risk of death at transplant. Morbidity varies greatly in the congenital patient population with the failing Fontan circulation having a far higher risk than a failing Mustard circulation. However the underlying issue of imbalance between donor organ supply and demand needs to be addressed as transplant teams are finding themselves in the increasingly difficult situation of supporting growing numbers of patients with a diverse range of pathologies with declining numbers of donor organs.

## BACKGROUND

The surgical technique of cardiac transplantation was first described in 1960, followed seven years later by the first successful human heart transplant. The procedure has subsequently evolved to provide a hugely important treatment modality to manage cardiac conditions which have either exhausted medical management strategies or would otherwise prove terminal. Although transplant is a valuable treatment, with the benefits of improving quality and duration of life, it is does not offer a cure in the conventional sense as it is currently unable to restore a fully normal life expectancy. The use of transplant is restricted by rigorous selection criteria of the transplant programme, assessment of the presenting condition, underlying pathology and co-morbidities being essential for full evaluation of the patient as to their suitability for transplant. The programme is limited however by the availability of donor organs, resulting in an increasing mismatch between supply and demand within the transplant service. As a result even with stringent assessment of those deemed suitable for transplant, additional consideration must be made to finding the most suitable recipient for the scarce and precious donor organ when it does become available. 

Conditions for which transplant may be an option are similar in both adult and paediatric populations, however the proportions of each presenting condition understandably are very different. In general heart transplantation is considered in individuals with end stage heart failure, with a life expectancy of less than 18 months, where other medical therapy has been exhausted or ineffective. Heart transplant is generally offered to patients who are under the age of 60 years. There are absolute and relative contraindications to cardiac transplantation, including significant medical, infective and psychiatric co-morbidities in addition to physiological status at the time of assessment, however there are no UK or international guidelines specifically relating to patients with congenital heart disease (See Appendix A). In children with congenital heart disease the general indications for transplant are the same as for other patient groups described above. More specific situations where cardiac transplant is appropriate include pulmonary atresia with intact septum, heterotaxy syndromes with anomalous pulmonary venous return and severe valve disease [1].

Approximately 1 in every 145 births in the UK is affected by congenital heart conditions [2] some are antenatally diagnosed but the majority are not detected until after delivery. The congenital defects arising may be minor, requiring no or minimal intervention, others are more severe and need graded management, either medical or surgical. With improving diagnostic, neonatal, intensive care and surgical skills increasing numbers of children with more severe lesions are able to survive childhood and into adult life. 

Of the 4,600 babies born with congenital heart disease every year in the UK [3] approximately 800 undergo surgical procedures in the neonatal period, 1500 within the first year and a further 1500 within the first 16 years of life [4]. These procedures may be reparative, staged or palliative depending on the underlying diagnosis and are associated with varying consequent degrees of morbidity and mortality. 

In the UK during the period 2009 to 2010 120 heart transplants were performed, of which 40 were in children (heart-lung transplant data for the same period being 5 in adults and none in children) [5]. Worldwide of the infants transplanted approximately two thirds had congenital heart disease whereas over one year of age this proportion reduced to only a third [6]. In congenital heart disease heart transplant may be offered as a primary management or offered after palliative or reparative surgical attempts either acutely or after a period of gradual failure.

## HEART TRANSPLANTATION IN PATIENTS WITH CONGENITAL HEART DISEASE

The majority of patients with congenital heart disease who receive a heart transplant do so after other surgical interventions have been attempted, either palliative or reparative. Those who receive a transplant as a first line management usually do so in early life, with subsequent peaks of transplants occurring in the 6 month to 6 year age group, adolescents and 20-40 year age group, reflecting the natural course of the underlying pathologies [7]. Increasing numbers of patients with congenital heart defects are surviving as a result of improved surgical and medical techniques as well as intensive care services. This results however in a larger number of patients presenting outside the period of infancy with end stage heart failure, requiring transplantation. The expanding congenital patient group placing extra strain on this already stretched resource. 

## THE USE OF HEART TRANSPLANTATION AS FIRST CHOICE TREATMENT

Heart transplants used as the first line management option for patients with congenital heart disease are usually performed in infancy. As a treatment modality it is deemed appropriate to offer the opportunity of transplant if the congenital lesion is not suitable for other surgical intervention or if the risk of other management, including surgery, is greater than that of transplant itself. On this basis transplant for patients with hypoplastic left hearts was advocated early in the history of paediatric cardiac transplantation when the outcome of palliative procedures was less successful. The early positive results seen as a result of transplant in these patients demonstrated its possible utilisation in the management of other cardiac conditions requiring intervention in early life and resulted in its wider use in the management of other congenital cardiac lesions, disease and cardiomyopathies of infancy. The outcome of staged palliative surgical procedures for hypoplastic left heart has subsequently improved and as a result the use of transplant as first line management in hypoplastic left heart disease has decreased [7]. 

The early experience of the newborns with hypoplastic left hearts has resulted in the wider use of transplant in infancy for other pathologies. As a consequence the infant donor pool has remained relatively constant. Congenital lesions that are now considered for heart transplant as a first choice management include hypoplastic left heart with abnormalities which preclude the Norwood operation (eg impaired ventricular function), severe Ebstein’s anomaly, pulmonary atresia with intact ventricular septum associated with abnormal coronary anatomy, severe valve abnormalities and heterotaxy lesions 

Transplantation in infancy appears to confer an immunological advantage long term, as infants do not produce ABO antibodies for the first few months of life and so their risk of hyper-acute rejection related to ABO incompatibility is reduced [8]. ABO mismatched transplants, usually contraindicated in older patients, can therefore be performed in this group increasing the potential donor pool. The age limit for such mismatched transplants is generally determined by measurement of circulating isohaemagglutinins in the individual [9]. The risk of rejection related to HLA antibodies remains unchanged and is discussed later.

Post transplant coronary artery disease is also seen less in infants, possibly related to infection exposure of both the donor and recipient. 

## THE USE OF HEART TRANSPLANTATION AFTER ONE OR MORE SURGICAL PALLIATIONS

Heart transplantation may be required after previous cardiac surgical procedures, many operations performed for patients with congenital heart disease not being curative, and ultimately resulting in heart failure after variable periods of time. A patient may be referred for transplant acutely if peri-operative complications are encountered, or electively after a longer period of management following a corrective or palliative procedure. 

Surgery performed on patients with congenital heart conditions may be complicated by associated defects such as abnormal situs, presence of collaterals and atypical vasculature. Unusual anatomy makes accurate transplant assessment of this patient group more difficult. Pulmonary vascular resistance (PVR) needs to be assessed prior to transplant to avoid later post transplant right heart failure in the transplanted heart [10]. PVR measurement is used as an indication of feasibility of transplant, high fixed PVR a contraindication to orthotopic heart transplant, although heterotopic or heart-lung transplants may still be an option.

In some cases the PVR is elevated post Fontan procedure. The failing Fontan is a difficult situation where collaterals and intracardiac shunts make accurate estimation of the PVR complicated. Fontan circulation deterioration may present as either ventricular failure with a normal PVR or with high venous pressures and relatively preserved cardiac function, the latter is usually related to high PVR. Failing Fontan physiology may be demonstrated clinically by protein losing enteropathy, ascites, plastic bronchitis and pleural effusions [11]. The consequences of this abnormal physiology are significant and elevate the mortality risk in patients who subsequently receive a transplant, the high PVR increasing the probability of right heart failure post transplant. Transplant for the single ventricle appears to be at much lower risk if undertaken at the stage of systemic or venous shunt rather than completed Fontan [12]. 

## REFERRAL FOR ASSESSMENT BY A TRANSPLANT TEAM

Early transplant assessment of any patient but particularly those with congenital heart disorders is clearly prudent [13]. Assessment provides an opportunity to evaluate the patient and institute management measures to reduce the impact of co-morbidities or interventions which may later limit their consideration for heart transplantation. Alternatively, after evaluation when non-transplant based options are deemed more appropriate, palliative and supportive measures can be discussed and instituted early as short or long term care strategies.

## ASSESSMENT

The transplant assessment is a dynamic process involving physiological and psychological determination of parameters regarding the potential recipient which may indicate short and long term, pre, peri and post operative complications for consideration. 

## HISTORY

A full previous medical history must be taken from the patient and allied professionals and medical records providing details of previous surgical interventions or cardiothoracic procedures that may help evaluate the previous anatomy and condition of the recipient. Surgical procedures confer different risks for any subsequent transplant to the individual patient through sensitisation, increased risk of bleeding, infection, wound dehiscence and adhesions. Previous surgery may have implications of later complications at transplant surgery. Repeat sternotomies may increase the risk of re-entry injury however data seems to suggest that this is not associated with increased risk of subsequent morbidity or of peri-operative mortality, it should however remain a factor for the operating team to be aware of [14, 15]. Previous thoracotomies may also increase the risk of significant adhesions and peri-operative bleeding at later surgery or transplant. 

Prior surgical intervention indicates the potential previous exposure of the patient to blood products and human tissue homografts, an additional aspect of the patient’s history which must be fully evaluated prior to listing as use of such products increases the problem of immunological sensitisation of the patient, an issue that will be discussed later.

Those who have undergone earlier cardiac surgery may also be left with atypical anatomy and communications that necessitate technical modification of the usual transplant procedure. 

## EXAMINATION

Classically older patients with heart failure that are being considered for heart transplant present with symptoms of cardiogenic shock, shortness of breath, reduced exercise tolerance, anorexia and weight loss. In children signs may be more subtle and difficult to interpret, poor feeding, vomiting and lethargy not being uncommon findings within the paediatric setting, but growth failure generally providing a good warning sign that further investigation is warranted. 

## EXERCISE TESTING

Cardiopulmonary exercise testing is frequently used to evaluate patients prior to transplant. This assessment tool is mainly used in adult patients and some older paediatric patients, the information obtained contributing to the overall evaluation of the condition of the potential transplant recipient. The data obtained provides an indicator measure of morbidity and mortality relative to age and sex, although definitive statistics are difficult to obtain regarding the implication of exercise testing results as numerous exercise protocols exist. Oxygen consumption during a period of exercise is measured, and also commonly in the UK a six-minute walk test is performed, but increasingly additional parameters are being considered as equal if not more reliable indices of assessment. Reduced autonomic response to exercise as determined by minimal heart rate elevation in response to an exercise challenge is associated with a higher risk of mortality in adult patients with congenital heart disease in general [16]. Additionally the assessment of metabolic function by measurement of carbon dioxide production relative to ventilation is increasingly being considered as an additional and significant prognostic variable [17]. The usefulness of exercise tolerance results after cardiac surgical procedures is uncertain. It has been shown that after certain procedures such as the Fontan procedure, exercise tolerance may never be normal and is seen to gradually decline further, therefore sub-optimal results may be expected which makes it hard to give definitive values regarding timing of transplantation.

## ECHO/ MRI

Echocardiogram is the most widely used imaging modality in cardiology, and it is a valuable tool in the assessment of potential transplant patients. Increasingly the more refined three dimensional imaging obtained by cardiac magnetic resonance imaging is being utilised to add to pre-operative assessment of patients. This imaging modality provides the clinician with invaluable additional data regarding structure, function, failure, tissue mass and fluid flow of the cardiovascular system. The relationship of any conduits to the sternum and the venous connections should be determined. The pulmonary arteries need to be carefully imaged to allow a planned repair of these at transplant if needed. However, it is well known that pulmonary artery reconstruction is an important risk factor for transplant [18]. 

## BNP

Natriuretic peptides (NP) are a group of hormones responsible for the regulation of sodium and fluid balance within the body. Of the three major classes of natriuretic peptides, A, B and C, A type natriuretic peptide (ANP) and B type natriuretic peptide (BNP) are released predominantly by the heart, their levels alter measurably in the blood in response to the mechanical stress placed on the myocardium. 

BNP is a valuable prognostic marker used in the assessment of heart failure. A strong association between BNP levels has been demonstrated with echocardiogram indices, clinical status and prognosis in children with left ventricular failure [19]. The elevated BNP identifying children at high risk of poor outcome in terms of prognosis and additional morbidity [20, 21]. 

In adults a single BNP measurement in patients with chronic heart failure may similarly identify those patients at highest risk of death, and may be a better predictive indicator than measurement of left ventricular ejection fraction by echocardiogram, peak oxygen uptake on exercise testing or composite scoring systems such as heart failure survival score [22, 23]. However, it is unclear whether BNP is useful in the transplant assessment of complex congenital heart disease. BNP appears to be of no clinical or diagnostic value in patients with a systemic right ventricle or Fontan circulation [24] and is also not elevated in failure of cavopulmonary circuits with preserved ventricular function [25] but may vary with systemic ventricular morphology. As a result it is unlikely to be as useful in isolation in assessment of congenital heart disease as it appears to be in simple left ventricular failure, but may be helpful as part of a systematic assessment [26].

## SENSITISATION

A matching system using human leucocyte antigens, those proteins on the cell surface that enable the host to differentiate self from non-self, is used in other solid organ transplant protocols. The aim being to have as close a match in these presenting proteins as possible to reduce the likelihood of transplanted organ rejection by the host. Although it is known that as few as three HLA mismatches may result in greater predisposition to rejection, unlike in other organ transplant programmes, full cross matching is not possible prior to transplant because of the limited supply of organs and an ischaemic time that must be kept to a minimum. A pre-transplant screening of the anti-HLA antibodies of potential recipients is however performed using a panel of antibodies sensitised to those most common HLAs. Panel Reactive Antibody (PRA) screening provides the transplant team with an indication of the potential immunological difficulty in finding a suitable donor organ, and some insight as to the extent of immunomodulation therapy that will be required to either empirically prepare a patient for transplant or manage the patient post-operatively. Patients with congenital heart disease are more likely than most other potential heart transplant recipients to be exposed to blood products or human tissue during their previous management. The increasing use of mechanical cardiac support and left ventricular assist devices, requiring frequent blood product transfusion also primes the recipient against HLAs. Such exposure of the patient to foreign HLA proteins greatly magnifies the risk of pre-transplant sensitisation, reducing the probability of finding an immunologically good tissue match at transplant. Special precautions are therefore required to deal with the likelihood of sensitisation in this patient group. Combating this higher risk scenario requires directed immunotherapeutic strategies pre- and post transplant, with a considerable risk of early rejection a consequence if they are not adequately controlled. 

## END ORGAN DAMAGE

With a failing circulation cardiac output is inadequate to meet the requirements of other vital organs. Resultant end-organ damage is frequently encountered and may be either reversible if addressed early or irreversible if refractive to therapy and intervention. With patients suffering with congenital heart disease surviving longer into adult life, additional complications and co-morbidities are being increasingly observed as a result of end organ damage. The risk of end organ damage may be age-related, especially in patients with congenital heart disease as increased duration of exposure to a sub-optimal circulation and the risk of additional organ damage is more predictable. With the limited supply of transplantable hearts it is vital the potential host is assessed to establish the severity and reversibility of such damage. Although transplant may be the only treatment modality to extend life in certain patients, it is inappropriate to transplant a new organ into a failing body that will not be able to recover sufficiently its other vital functions. Even if additional organ failure is severe it may be reversible if provided with a restored and appropriate circulation, it is therefore the reversibility of the end organ damage that is most important for transplant teams to assess but difficult to predict. 

All organ systems will be affected to some extent when in an environment of a failing circulation. Renal dysfunction is commonly seen as a consequence of heart failure. Approximately half the adult patients with congenital heart disease, particularly those with cyanotic conditions, have been observed to have significantly impaired renal function [27]. A three fold increase in death rate has been seen in those patients with moderate to severe renal failure than in patients with normal kidney function [27]. 

The pathophysiology of renal failure seen in patients with congenital heart disease is likely to be similar to that seen in heart failure acquired secondary to other conditions. Low cardiac output causing chronic renal hypoperfusion results in neurohormonal and autonomic upregulation measures in an attempt to restore an appropriate circulatory pressure. This unsustainable situation culminates in renal damage quantifiable in terms of blood analysis and GFR measurement.

Persistent hyponatraemia appears to be an indicator of outcome in patients with heart failure, independent of haemodynamic status [28]. 

Anaemia is seen in adults with congenital heart disease, and may be associated with a three- fold increase in the risk of death [29]. The pathophysiological process causing the anaemia being reduced erythropoietin production secondary to renal dysfunction. This is compounded by anaemia of chronic disease, seen as a consequence of acute or chronic immune activation. Although some aspects of this anaemia can be corrected, by administration of erythropoietin, iron supplementation and other measures, its presence itself indicates the severity of the underlying disease process and is highlighted through the presenting clinical condition of the patient. The anaemia results in reduced oxygen carrying capacity and a transfer to anaerobic metabolism at an earlier stage than should be normally seen, and is recognised by a reduced exercise tolerance and an abnormal ventilation-carbon dioxide production relationship on metabolic testing. Polycythemia is seen in severely cyanotic patients and can increase the risk of surgery and bleeding is more common in these patients post operatively.

Liver disease is also observed in patients with end stage cardiac failure, the hepatic dysfunction seen causing abnormal clotting and fibrosis, later leading to cirrhosis. Liver status clearly being an important factor in pre-transplant assessment. This is a particular problem with the failing Fontan circulation [30].

## SUBJECTIVE ASSESSMENT- QUESTIONNAIRE

Both subjective and objective evaluation of the potential transplant patient is important. The use of the Minnesota Living with Heart Failure self assessment questionnaire is frequently used in adult heart failure patients. Other similar self- assessment surveys also exist focusing on additional specific aspects of heart failure, such as the London Chest Activities of Daily Living Scale. The wording and interpretation of the questions by patients in self evaluation does however result in variable reliability in the data that it is intended to assess [31]. Self estimation by patients of their physical functioning has been seen to relate poorly to their actual exercise capacity [32], but such questionnaires still provide valid information to support other more objective investigations. 

## PSYCHOLOGICAL AND NEUROLOGICAL STATUS

The psychological stability of patients being assessed for transplant is also reviewed. Adults with chronic psychiatric illness or substance dependence may be deemed unsuitable for the transplant process. The wait for transplant once listed is also an extremely difficult time for all patients and their families so ongoing support and assessment should be maintained. Poor quality of life and inability to participate in the normal routines and activities of daily living affect the emotional well being of children and adults alike. Patients with congenital heart disease frequently only rate their physical functioning and general health during assessment, rarely ranking psychosocial aspects of their illness, despite observations that even minor symptoms of depression exert a more significant impact on their quality of life than exercise capacity [32, 33].

The neurological status and development of patients with congenital heart disease is also pertinent. Neurological complications of uncorrected congenital disorders include stroke and brain abscess, as well as the adverse influence on neuro-development. In addition there are complications that arise as a consequence of cardiac surgery especially in the young, the neurological complications of cardiac bypass and hypothermic circulatory arrest being well documented [34]. 

## MECHANICAL SUPPORT

When the cardiac status fails completely and mechanical support is required to preserve life, the risk of additional complications is magnified both through the severity of the presenting heart failure and the risks associated with its management. 

Extra-corporeal membrane oxygenation (ECMO) may be more rapidly utilised than left ventricular assist devices and may provide short term support in emergency situations, but carries considerable procedural and consequent risk. It has been more commonly used in paediatrics than in adults. It is clear that ECMO significantly increases the risk of heart transplantation. Left ventricular assist devices however offer the patient the advantages of long term cardiac support without the need for continuous sedation or respiratory support, indeed many patients live relatively normal lives with an LVAD inserted whilst waiting the increasingly long periods for a transplant. Studies have concluded that children requiring LVAD support prior to heart transplant have the same survival outcomes as those who do not [35]. 

However, there is very little in the literature on VAD support for failing congenital heart disease. It is clearly difficult to balance pulmonary and systemic flow in the failing single ventricle and may be very difficult in the failing Fontan circulation. There are some successful case reports, but the wider US experience with the Berlin Heart which will be published in the near future will give a much clearer idea about the risk of support in complex congenital heart disease. 

The need for ventilator support prior to cardiac transplant also confers poorer prognostic outcome. 

## OUTCOME OF ASSESSMENT

Rigorous assessment by the multi-disciplinary team enables the most appropriate candidates to be selected for heart transplantation. Patients may be categorised on the results of the described investigations as being either high or low risk for the procedure and are also classified as either urgent or standard depending on their prevailing clinical condition. Some patients with congenital cardiac defects who do survive childhood and progress to adult cardiac services may not however get listed due to the consequent high risk presentation of end organ damage, raised PVR, previous thoracotomies and associated adhesions, chronic formation of collateral circulations and immune sensitisation. 

Due to the shortage of donor organs priority is generally given to the sickest patients, with those individuals listed as urgently needing a new organ being considered before others that are more stable when an organ becomes available. The psychological strain on the individual and the family involved whilst waiting for an indeterminate duration on a transplant waiting list is hard to comprehend The clinical status of the patient may fluctuate during this time and due to the supply and demand inequality of the transplant programme, patients die whilst waiting for a new heart. In the United States of all the children listed for heart transplantation between 1999 and 2006 17% died before an organ became available for them [36]. 

## ENVIRONMENT NEEDED TO PROVIDE A RELIABLE SERVICE

To optimise the effectiveness of a cardiac transplantation programme for patients with congenital heart disease facilities and skills need to be appropriate for the difficulties that may be encountered. The available surgeons and transplant team including nurses, anaesthetists, perfusionists, cardiologists and intensivists need to be skilled in the field of congenital cardiac surgery themselves, so that atypical anatomy, previous reparative surgical techniques and potential hazards may be appreciated and anticipated. Donor organs for patients with congenital defects often require extended structures to remain intact at explantation to permit effective transplantation into the recipient with structurally abnormal anatomy. Extended harvest of systemic and pulmonary vasculature may assist with the implantation into the recipient but may limit the use of other potential donor organs, particularly the lungs. Consideration needs also to be paid to managing the medical risk factors and complications highlighted such as renal dysfunction, clotting aberrations, elevated PVR and sensitisation. 

## POSSIBLE SOLUTIONS

At a critical time for the heart transplant service, as donor organs are in such low supply, but demands in all age groups remain high, serious thought must be given as to how we may redress the balance. More rigorous assessment criteria could be imposed restricting the use of the limited organs offered to those with the best potential outcome, but this would limit the service by exclusion of whole patient populations, including those with congenital lesions. But not all congenital heart disease is the same. For example transplant following the Senning and Mustard procedures is possible with low risk, while transplant for the failing Fontan patients has an 8.6 times increased relative risk of death [37]. Careful consideration of multiple risk factors can give an estimate of the potential morbidity and mortality of cardiac transplant in an individual, some patients may be considered so high risk that transplant is not a viable option. For example a recent post sternotomy Fontan patient on ECMO, with impaired renal function, probable high PVR, protein losing enteropathy and high immunological sensitization would be a very high risk case, whereas an ambulatory, non-sensitised Senning patient with systemic ventricular failure would be a low risk case.  Exactly where to draw the line between these two extremes has not been defined, yet as organ availability declines transplant physicians will have to make very difficult decisions for the congenital heart disease patients.

With the advantages of heart transplant in infancy observed, perhaps more transplants should be aimed at this age group, even if organs are not best matched immunologically and may even be marginal in other characteristrics such as size, good outcomes are still observed. Heart transplantation in infancy may be advantageous in many ways but may also enforce the life- limiting implications of transplant onto the extremely young before the natural course of the disorder can be fully determined. Ethical debate persists as to how donors may be most appropriately selected and creates another topic for crucial negotiation [38]. 

As heart failure management progresses, particularly with the increasing use of mechanical support in all age groups, perhaps clinicians will be focusing on alternative and novel treatment modalities to reduce the need of transplant, especially for conditions where a structurally normal heart exists, but where dysfunction has occurred via disease processes such as muscle damage. Reducing the demand for organs by medical or surgical means would begin to modify such an unbalanced situation.

## SUMMARY

The field of cardiac transplantation is clearly complicated with additional early risk factors for morbidity and mortality attached than are encountered in many other transplant situations, but with careful planning these may be addressed and minimised. If patients survive the early operative complications of their transplant, late survival rates appear to be comparable to survival of transplant for other cardiac indications such as cardiomyopathy [39]. This trend is seen in both adults and children requiring transplant for congenital heart disease [39, 40]. 

Many factors contribute to the decision to transplant patients with congenital heart disease. Consideration should be paid to the optimal timing of referral to a transplant team for assessment. Assessment of paediatric and adult patients varies considerably in ease of assessment and reliability of results. Certain clinical presentations and underlying diagnoses as well as co-morbidities are recognised as stratifying patients to high risk and low risk categories for outcome of transplant, indeed certain pathologies are deemed to be contraindications to transplant. The ISHLT has brought together experts in the field of heart transplantation from around the world to develop consensus guidelines for the management of transplantation. Their guidelines offer advice regarding suitability of donor organs and peri-and post operative management. 

With increasingly short supplies of organs available for transplantation, and those being donated often crossing international boundaries, it is more important than ever that transplant teams, clinicians and co-ordinators maintain communication to produce and update structured guidelines and international standards on which informed and rational decisions can be made as to how these organs can be most effectively utilised. The complex and continually evolving field of transplantation, particularly for congenital heart disease, deserves international collaboration *via* consultation and database registries to permit effective management of the individual patient. Difficulty arises however when attempting to provide a unified management strategy when clearly the field of congenital heart disease is such a heterogeneous topic, with variability in risk factors, co-morbidities and outcomes [13] yet it is incumbent on transplant teams to begin to address this difficult subject.

## Figures and Tables

**Table 1:** Common Underlying Diagnoses by Age in Patients Requiring Heart Transplant

Age	Diagnosis	Further Details

< 1 month (infancy)	Hypoplastic left heart with abnormalities precluding Norwood procedure	Impaired ventricular function and inlet valve regurgitation
	Severe Ebstein’s anomaly	Symptomatic infants
	Pulmonary atresia with intact ventricular septum and abnormal coronary anatomy	Right ventricular dependent coronary circulation
	Severe valve abnormalities	eg mitral regurgitation with impaired function.
	Heterotaxy lesions	Impaired ventricular function and inlet valve regurgitationf

>6 months	Single ventricle	At various stages of palliation eg Blalock shunt (infants), Glenn shunt (young child), Fontan (older child)
	Transposition of great arteries	Typically post Mustard or Senning
	Right ventricular outflow tract lesions	eg post op. repair with impairment of ventricular function
	Ventricular/atrial septal defect	eg post op. repair with impairment of ventricular function
	Left ventricular outflow tract lesions	eg post op. repair with impairment of ventricular function
	Congenitally corrected-transposition of the great arteries	eg post op. repair with impairment of ventricular function. Sometimes post double switch operation
	Complete atrioventricular septal defects	eg post op. repair with impairment of ventricular function and severe valve regurgitation

## Appendix A: Contraindications to Heart Transplantation

( Data taken from UK Transplant National Protocol for Assessment of Cardiothoracic Transplant patients, published March 2002, reviewed February 2005, prepared by the UKT Cardiothoracic Advisory Group):

**Table:**

Absolute Contraindications to Heart Transplantation:	Relative Contraindications to Heart Transplantation:
Chronic current systemic infection, including endocarditis Chronic extracardiac infection Active peptic ulcer Continued abuse of alcohol or other drugs Irreversible secondary organ failure unless considering for combined transplant Psychiatric history likely to result in non-compliance and/ or persistent non-compliance with medical therapy Severe peripheral or cerebrovascular disease Malignancy Other life-threatening medical condition, likely to cause death within five years	HIV ( subject to discussion with Medical Director at UK Transplant) Hepatitis B/C Acute pulmonary embolus ( within 3 months) Obesity BMI>30 COPD with FEV1<50% predicted Pulmonary vascular resistance greater than 4 Woods Units Transpulmonary gradient greater than 12mmHg Chronic renal impairment with GFR<50ml/min, unless candidate for combined renal transplant Diabetes with target organ damage Hypercholesterolaemia or other lipid diseases refractory to diet or drug therapy Severe osteoporosis (bone mineral density > 2sd’s less than predicted for age) Amyloidosis Continued smoking Giant cell myocarditis
